# Epidemiology of primary rubella infection in the Central African Republic: data from measles surveillance, 2007–2014

**DOI:** 10.1186/s12879-016-1842-2

**Published:** 2016-09-23

**Authors:** Alain Farra, Marilou Pagonendji, Alexandre Manikariza, Dieubéni Rawago, Rock Ouambita-Mabo, Gilbert Guifara, Ionela Gouandjika-Vasilache

**Affiliations:** 1 Enteric viruses and Measles Laboratory, Institut Pasteur de Bangui, Bangui, BP 923 Central African Republic; 2 Epidemiology Unit, Institut Pasteur de Bangui, Bangui, Central African Republic; 3 Expanded Programme on Immunization, Ministry of Health, Bangui, Central African Republic; 4 Epidemiological Surveillance Unit, World Health Organization, Bangui, Central African Republic

**Keywords:** Rubella, IgM, Sero-prevalence, Central African Republic

## Abstract

**Background:**

Although rubella is generally considered a benign childhood disease, infection of a pregnant woman can cause foetal congenital rubella syndrome, which results in embryo-foetal disease and malformations. The syndrome is still a public health problem in developing countries where the vaccine has not yet been introduced, such as the Central African Republic (CAR). The aim of the study reported here was to define the epidemiology of primary rubella infection, in order to determine its effect on morbidity rates in the country.

**Methods:**

Data derived from epidemiological surveillance of measles and rubella were analysed retrospectively between 1 January 2007 and 31 December 2014. The database includes cases of suspected measles, according to the WHO clinical case definition. In this algorithm, samples that are negative or doubtful by ELISA for measles (presence of immunoglobulin M) are tested in another ELISA for detection of rubella-specific IgM. Descriptive analyses were conducted for socio-demographic characteristics, including age, sex and health region, for patients tested for rubella.

**Results:**

Of the sera tested for rubella, 30.2 % (425/1409) were positive, 62.3 % (878/1409) were negative, and 7.5 % (106/1409) were doubtful. Among the 425 positive cases, 213 (50.1 %) were female and 212 (40.9 %) were male with a sex ratio of 1.03. The mean age was 8 years (range, 6–37 years). The highest prevalence (47.3 %; 116/425) was seen in 2007 and the lowest (8.9 %; 11/425) in 2012. Primary infections were always more frequent during the first 3 months of the year, with a peak at the same time, between January and February which is the hottest period of the year in the CAR. In both sexes, rubella IgM was rarely found before the age of 1 year (0.5 %; 2/425). The highest rate (43.5 %; 185/425) was observed at ages 5–9 years; however, at least 8 % (18/213) of girls aged 15 or more had primary infections.

**Conclusions:**

Sentinel sites for surveillance of congenital rubella syndrome are urgently needed, and introduction of vaccination against rubella in the Expanded Programme of Immunization should be considered, to ensure immunization of girls of reproductive age.

## Background

Rubella is an infectious disease caused by an RNA envelope virus of the *Togaviridae* family and the *Rubivirus* genus. This strictly human virus, which is transmitted in aerosols, usually causes a benign infection in children and young adults. Its public health importance is due to the mother’s risk for congenital rubella syndrome after a primary infection at the beginning of pregnancy. The syndrome is associated with embryo and foetal disease and congenital malformations [1, 2].

The prevalence of primary infection is difficult to determine, but there are an estimated 110 000 cases of congenital rubella syndrome each year in developing countries, even though this is a vaccine-preventable disease and rubella is a viral disease that could potentially be eradicated [3, 4].

The prevalence of infections by specific rubella IgM detection reported by the World Health Organisation (WHO) for the Africa region between 2002 and 2009 ranged from 13 and 38 % [1]. Evaluations of the surveillance of rubella infection in Ethiopia between 2004 and 2009 and in Nigeria in 2011 have respectively reported a prevalence of 12.1 and 45.2 % [5, 6]. In Central African Republic (CAR), a study on the seroprevalence of natural measles and rubella antibodies in children under 15 year, based on the detection of IgG in Bangui had reported in 2008 an overall prevalence of 55.4 % for rubella [7]. But no studies had yet dealt with the assessment of the primo infection based on the detection of specific IgM.

The expanded programme of immunization established in CAR by the Ministry of Health in 1979 with the support of the World Health Organization (WHO) and UNICEF still does not include rubella vaccine. Measles vaccine is administered at the age of 9 months, with an estimated coverage of 50 % in 2014 and 33 % in the first 3 months of 2015; these rates are too low for WHO to consider introducing rubella vaccine in the CAR [2]. Although the Institut Pasteur de Bangui commercializes the trivalent vaccine against measles, mumps and rubella, few children have access to it because of its high cost: in 2007–2014, only 369 children were vaccinated, corresponding to about 50 per year. Therefore, children are generally immunized naturally, by primary infection; when a pregnant woman is infected, is at risk for congenital rubella syndrome [8].

Epidemiological surveillance of measles and rubella has been conducted in the CAR since 2007. The samples that are negative for measles are systematically tested for primary rubella infection by differential diagnosis according to the WHO-recommended algorithm [1, 9]. The enteric viruses and measles laboratory at the Institut Pasteur de Bangui is a WHO national reference laboratory for measles and is the only structure for sero-surveillance in the country. In this study, we evaluated the epidemiology of primary rubella infection between 2007 and 2014 in the CAR.

## Methods

### Study site and demographic data

The CAR, in the heart of the African continent, covers an area of 623 000 km^2^ and has an equatorial climate, with two seasons: a rainy season between May and October and a dry season between November and April. The population, which was 3 895 139 in 2003, is estimated on the basis of a natural annual growth of 2.5 % to be 4 200 854 in 2007 and 4 854 905 in 2014. The regional distribution of this mainly rural population (61 %) results in a density of 11 people per km^2^ in the west and < 1/km^2^ in the east; in the centre, the south and the south-east, the density varies from 7.2/km^2^ to 10 041.38/km^2^ in the capital, Bangui [10]. The health system is decentralized into seven regions to ensure effective coverage with health programmes.

### Database, target population and sampling

Data from sero-surveillance of measles and rubella between 1 January 2007 and 31 December 2014 were analysed retrospectively. The Epi Info database includes information on the type of sample, its origin and sociodemographic data on the donors.

Patients who gave samples met the WHO standard definition of suspected cases of measles, i.e. generalized maculopapular rash and fever, with at least one of the following symptoms: cough, rhinitis or conjunctivitis; the samples were taken within 28 days of the beginning of the rash [1]. The samples consisted of 1–5 ml of venous blood drawn into dry tubes and transported in refrigerated sample carriers at 4 °C - 8 °C to the laboratory within 3 days of sampling. Urine samples and throat swabs, which might have assisted diagnosis, were not used.

Samples were taken in all seven health regions, and transport to the laboratory was ensured both by trained focal points in the health regions and by nongovernmental organizations such as Médecins sans Frontières and Médecins du Monde.

The data obtained for each patient were: age, sex, history of vaccination against measles, date of onset of clinical signs, dates of sampling and hospitalization.

### Laboratory analyses

First, the measles IgM research was performed on all samples. According to the algorithm defined by WHO, samples negative or doubtful for measles were tested for rubella in an indirect enzyme-linked immunosorbent assay (ELISA) for quantitative detection of specific rubella virus immunglobulin (Ig) M (Anti-rubella Virus/IgM Enygnost, Siemens). This test has a sensitivity of 98 % and a specificity of 97.3 %. The sensitivity and the specificity of the test have been made optimum by the initial treatment of Sera by the absorbing factor from the Kit, that forming an immuno-complex with the IgG to neutralize the rheumatoid factors in Sera. In accordance with the criteria of validity for the test, sera with an optical density < 0.10 were considered negative, 0.10–0.20 as doubtful and > 0.2 as positive. The results were entered into the Epi Info measles database.

### Data analysis

The data collected were treated and analysed with Epi Info version 7 software and Excel. Descriptive analyses were conducted for the sociodemographic characteristics age, sex and health region.

## Results

### Characteristics of the study population

One thousand four hundred nine persons were involved in this study. The average age of the test subjects was 8 years with extremes ranging from 6 months to 37 years, the male/female sex ratio was 1.03. The numbers of subjects living in urban areas was 1002/1409 (71.1 %) against 407/1409 (28.9 %) in rural areas.

### Prevalence

Between January 2007 and December 2014, 1409 sera were tested for rubella-specific IgM; 30.2 % were positive, 62.3 % negative and 7.5 % doubtful (Table 1). The highest prevalence was seen in 2007 (47.3 %), followed by 2014 (37.7 %) and 2009 (30.8 %). The lowest prevalence was seen in 2012 (8.9 %).

**Table 1:** Results by year for suspected measles cases and rubella serology, 2007–2014

Year	Suspected measles case	No. of sera tested in rubella	Rubella IgM		
			Positive	Negative	Doubtful
2007	256	245 (95.7 %)	116 (47.3 %)	111 (45.3 %)	18 (7.4 %)
2008	116	104 (89.6 %)	22 (21.2 %)	77 (74 %)	5 (4.8 %)
2009	119	107 (90 %)	33 (30.8 %)	69 (64.5 %)	5 (4.7 %)
2010	95	93 (97.9 %)	14 (15.1 %)	77 (82.8 %)	2 (2.1 %)
2011	158	84 (53.2 %)	15 (17.8 %)	64 (76.2 %)	5 (6 %)
2012	190	123 (64.7 %)	11 (8.9 %)	103 (83.8 %)	9 (7.3 %)
2013	380	165 (43.4 %)	30 (18.2 %)	115 (69.7 %)	20 (12.1 %)
2014	722	488 (67.6 %)	184 (37.7 %)	262 (53.7 %)	42 (8.6 %)
All	2036	1409 (69.2 %)	425 (30.2 %)	878 (62.3 %)	106 (7.5 %)

### Distribution of seroprevalence by health region

**Fig. 1:**
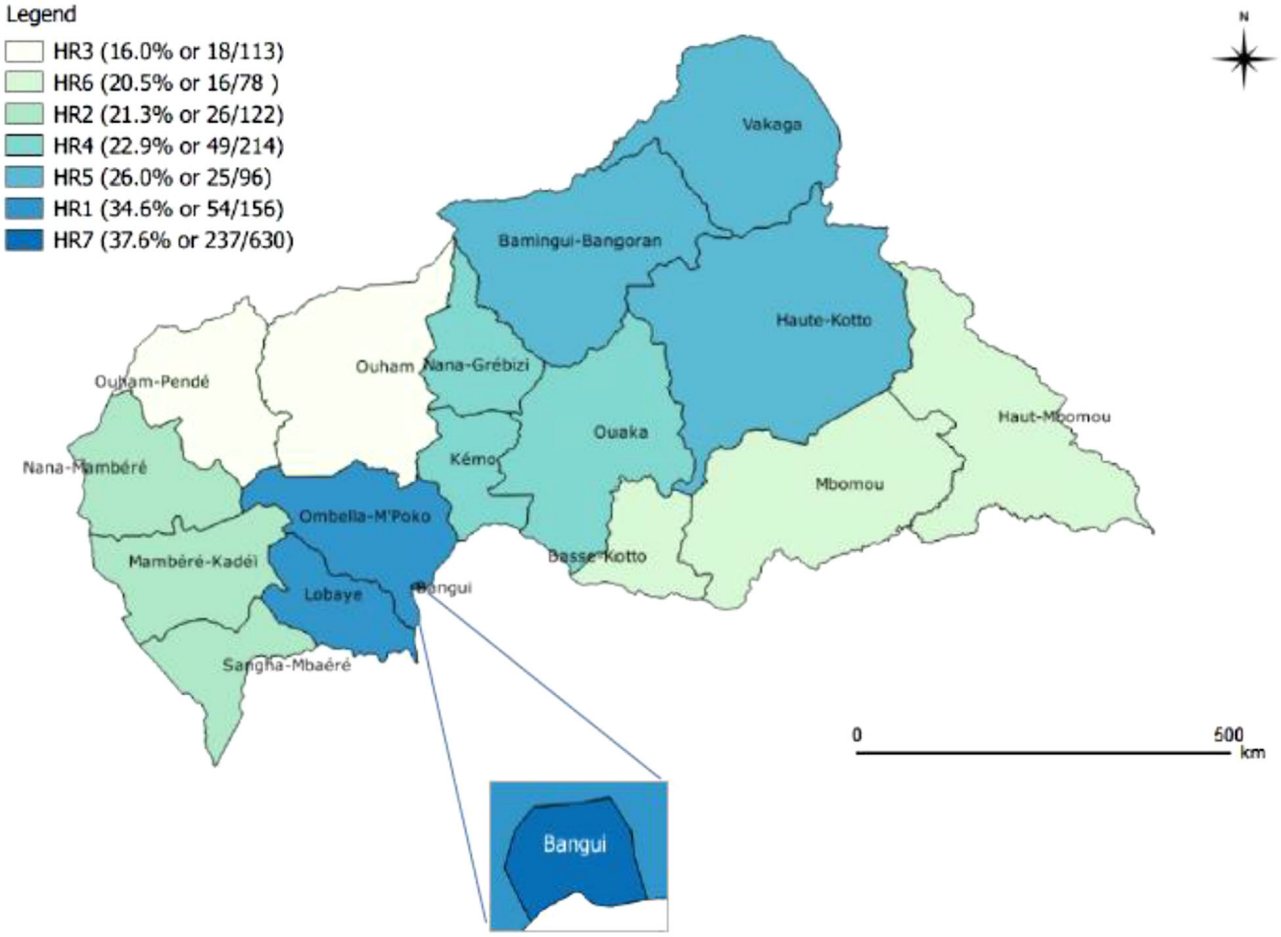
Distribution of rubella IgM by health region, 2007–2014

Figure 1 shows that laboratory-confirmed rubella was present in all seven regions, with the highest prevalence in health region 7 (237/630, 37.6 %), followed by region 1 (54/156, 34.6 %), and the lowest in region 3 (18/113, 16.0 %).

### Distribution of seroprevalence by month, 2007–2014

**Fig. 2:**
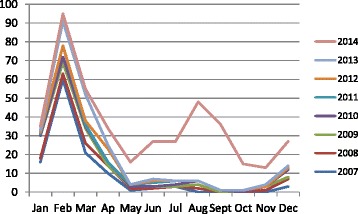
Distribution of cases of rubella by month, 2001–2014

Although cases are notified throughout the year, they occur mainly during the first 3 months, with a peak in January and February. In 2014, however, the notification rate remained higher throughout the year, with a second peak between July and August (Fig. 2). This peak affected particularly the region 7 where the number of positive IgM was 144 /184 (78.3 %).

### Distribution of seroprevalence by age and sex

Table 2 shows that rubella-specific IgM was present in equal proportions of girls (50.1 %) and boys (49.9 %). Rubella was rarely found before the age of 1 year (0.5 %) in either sex. Immunization begins from the age of 1 year, with a peak between 5 and 9 years (43.5 %). After the age of 15 years, at least 8 % of girls still had primary infections.

**Table 2:** Distribution of rubella IgM-positive samples by age range and sex

Sex	Age (years)					
	<1	1–4	5–9	10–14	≥15	All
Male	0 (0 %)	47 (22.2 %)	94 (44.3 %)	48 (22.6 %)	23 (10.9 %)	212 (49.9 %)
Female	2 (1.0 %)	36 (16.9 %)	91 (42.7 %)	66 (31.0 %)	18 (8.4 %)	213 (50.1 %)
All	2 (0.5 %)	83 (19.5 %)	185 (43.5 %)	114 (26.8 %)	41 (9.6 %)	425 (100 %)

## Discussion

Between 2007 and 2014, 425 laboratory-confirmed cases of rubella were found in 1409 samples from the seven health regions of the CAR, for a prevalence of 30.2 %; 62.3 % were negative and 7.5 % doubtful. These results are similar to those from laboratory surveillance in 40 countries in the WHO African Region, which showed a prevalence of 24.3 % (25 631/105 250), with 66 % negative and 4 % doubtful [1]. Although these data appear to be similar across countries, it is not clear whether they really define the epidemiological profile of rubella in Africa, as all the patients included had suspected measles [5]. The more than 60 % negative results obtained in this study and that of WHO for all countries might be due to the inclusion of children with other eruptive febrile illnesses of unknown aetiology, such as chicken pox, roseola, scarlet fever and iatrogenic skin rashes. Furthermore, samples taken very early, within 3 days of the start of illness, or late, at > 28 days after the start, might not have had high enough antibody titres to be detected serologically [5]. Monitoring such that it has been implemented in the African region of WHO relies solely on searching of specific IgM in sera from commercial ELISA kits. Since the 1980’s it is well known that IgM positive test is not enough to assert a primary rubella infection, because some viral infections like Epstein Barr virus infection (EBV), cytomegalovirus (CMV) or human parvovirus B19 (HPV-B19) can cause a cross-reaction with rubella infection [11–16]. This shows the limits of these surveillance programs in general and particular of this study, especially that IgG avidity tests were not performed to assert the primary rubella infection [15].

High primary infection rates were always seen during the first 3 months of each year, with a peak at exactly the same time, between January and February, which is the hottest period of the year. The influence of a hot, dry climate on peaks of infection and high disease prevalence were also reported in the USA before vaccination was introduced [17, 18]. High transmission during this period is associated with dry mucosa, with micro-lesions induced by breathing hot, dry air. The finding of the highest prevalence in health region 7 may be due to the high population density (10 000/km^2^), which would result in high transmission rates [17].

The primo infection was more observed in the urban population probably because in CAR, population lives preferentially in urban areas where the density is higher than in rural areas characterized by a disparate population. In other studies such as the one in Ethiopia, the population is rural to almost 83 % which may justify that a strong predominance is found in this environment [5].

Furthermore, during 2014 CAR experienced several military events that induced population movements all over the country, especially in region 7. Entire families found themselves in displaced camps which could promote the spread within groups and explain this peak between July and October.

The distribution of primary infection by age showed virtually no immunization before the age of 1 year (0.5 %), a rate of 19.5 % between 1 and 4 years, 43.5 % between 5 and 9 years, 28 % between 10 and 14 years and 9.6 % in children over 15 years. The data reported by WHO for the countries of the African Region are similar, with 3 % at < 1 year, 28 % between 1 and 4 years, 47 % between 5 and 9 years, 16 % between 10 and 14 years and 5 % in children over 15 years [1]. The low prevalence before 1 year of age may be due to passive immunization by maternal antibodies; children have primary infections after that age, with a peak between 5 and 9 years. This age range was associated with high levels of rubella infection even in developed countries, such as Australia, European countries and the USA, before introduction of the vaccine; children of these ages often spend time in high-density public places, such as schools, care establishments and leisure centres, which could contribute to propagation of infection [19].

We found little difference between the sexes, as in other studies [6, 20, 21]. This implies that, if vaccination were included in the expanded programme in the CAR, both sexes should be targeted in order to reduce circulation of the virus in communities [22]. In the absence of vaccination, 8.4 % of girls at least 15 years of age, who are of reproductive age, could expose their foetus to congenital rubella syndrome. The prevalence in the WHO African Region is 5 %, and a prevalence of 15–20 % has been reported in some studies [1, 22, 23]. The risk for congenital rubella is real, as demonstrated by the results of this study and in other African countries; however, lack of notification of cases, due to the absence of a system of surveillance, has concealed this fact [1]. Sentinel sites for the surveillance of congenital rubella syndrome should be established to evaluate the true burden among women of reproductive age in our countries, where immunization against rubella is still acquired randomly by primary infection.

## Conclusions

This retrospective study allowed us to determine the annual incidence and epidemiology of rubella between 2007 and 2014 in the CAR. Although rubella is a childhood disease, a substantial number of girls of reproductive age are exposed to primary infection and thus may transmit the virus *in utero*, with a risk for congenital rubella syndrome. Seasonality appears to be a key factor in transmission in the CAR and also the size of the population in which the disease occurs. We hope that these results will detrmine the Department of Health of the CAR and partners to use a better approach to epidemiological surveillance, particularly for congenital rubella syndrome. In the light of these results, the question of introducing rubella vaccine into the expanded programme should be addressed, while at the same time improving coverage with measles vaccine, in order to ensure that the children of the country are immunized against these two viral diseases.

## Abbreviations

CAR: Central African Republic
CMV: Cytomegalovirus
EBV: Epstein Barr virus
ELISA: Enzymz Linked Immuno Sorbant Essay
HPV-B19: Human Parvovirus B19
WHO: World Health Organisation
